# Impact of Dental Aesthetics on Dental Students’ Academic and Social Performance: A PIDAQ‐Based Study

**DOI:** 10.1155/ijod/9043993

**Published:** 2026-01-07

**Authors:** Aseel Sharaireh, Eman Hassuneh, Hiba Nasser, Nesreen Salem, Sanaa Aljamani, Hassan Kaabi, Faleh Sawair, Mohammad AL-Rabab’ah

**Affiliations:** ^1^ Department of Restorative Dentistry, School of Dentistry, The University of Jordan, Amman, Jordan, ju.edu.jo; ^2^ Department of Fixed and Removable Prosthodontics, School of Dentistry, The University of Jordan, Amman, Jordan, ju.edu.jo; ^3^ School of Dentistry, Zarqa University, Zarqa, 13110, Jordan, jadara.edu.jo; ^4^ Department of Oral Medicine and Diagnostic Sciences, College of Dentistry, King Saud University, Riyadh, Saudi Arabia, ksu.edu.sa; ^5^ Department of Oral and Maxillofacial Surgery, Oral Medicine and Periodontology, School of Dentistry, The University of Jordan, Amman, Jordan, ju.edu.jo

**Keywords:** academic performance, dental aesthetics, PIDAQ, social performance

## Abstract

**Objectives:**

The purpose of this study was to assess the effects of self‐reported perception of facial and dental aesthetics on academic and social performance among dental students in Jordan.

**Methods:**

Online and paper‐based cross‐sectional surveys were collected from 371 dental students distributed between the two dental schools in Jordan. The survey contained the five sections of the Psychological Impact of Dental Aesthetics Questionnaire (PIDAQ), then asked participants about the presence or absence of factors that compromise dental aesthetics, and information about academic scores and academic satisfaction.

**Results:**

Results showed that facial and dental aesthetics had an impact on the social and academic performance of dental students in Jordan. Students who stated perceived factors compromising their dental aesthetics reported low levels of confidence when speaking and communicating with other people, lower grades, and low levels of satisfaction with overall academic performance.

**Conclusions:**

Social and academic performance of dental students in Jordan were found to be significantly influenced by the aesthetic qualities of the self‐reported facial and dental features. These findings highlight potential psychosocial impacts of perceived dental aesthetics, while acknowledging that other unmeasured factors may also contribute.

## 1. Introduction

Various treatments such as teeth whitening, teeth realignment, direct and indirect veneers are used to address problems with dental aesthetics. An increasing number of university students are now seeking such aesthetic treatments for their teeth. This is thought to be due to the outburst of social media on the perception of aesthetics [1]. Moreover, young adults believe that their smiles can leave either a good or a bad first impression and thus they are more conscious of the impact of their dental aesthetics on social interactions [2–4].

The anterior teeth are more apparent when speaking, smiling, and laughing compared to the posterior teeth, thus their shape, colour, and alignment have a great impact on self‐satisfaction with appearance [5]. Tooth fractures, discolorations, caries lesions, malocclusions, gummy smiles, missing teeth, and broken crowns adversely affect the quality of smiles of individuals [6, 7].

It is documented that “good‐looking” students receive better academic evaluations than their less “good‐looking” peers [8–10]. Academic performance is the ability of the student to respond to learning stimuli and to reach previously established educational goals [11]. Most students aim to score high marks in their grade point average (GPA). This is due to several reasons, such as self‐approbation or competing for postgraduate positions. Nevertheless, most students identify their academic success based on their GPA scores [12].

Effective social communication is crucial for good academic performance [13]. It is of special importance for dental students who need to engage in effective communication with their patients, their supervisory teams, colleagues, and other health workers within the hospital where they are receiving their training. Dental students are known to pay more attention to a person’s teeth while communicating and thus are more critical in evaluating a smile due to their training [14].

High self‐esteem and self‐confidence are among the most important contributors to effective social communication [15] and are reported to affect individuals’ self‐perceived satisfaction with their smiles and oral health [16, 17]. Self‐esteem describes how a person values themselves and is one of the parameters of social performance. Studies have reported a significant correlation between dental aesthetics and self‐esteem, showing that decayed teeth and tooth loss negatively affected participants’ self‐esteem and psychological well‐being [6, 18, 19].

In this project, Dental students were selected as the study population because their academic and clinical training emphasises oral aesthetics, increasing their sensitivity to dental appearance both in themselves and in others. This heightened awareness distinguishes them from other student populations and has implications for their social interactions, professional communication, and academic performance. Understanding the psychosocial impact of dental aesthetics in this group has clinical and educational relevance, as it may inform interventions to enhance self‐confidence and well‐being among future dental professionals.

The Psychosocial Impact of Dental Aesthetic Questionnaire (PIDAQ) is a validated psychometric instrument containing 23 items [20]. Structurally, it is composed of four subscales, one positive and three negatives, which represent four domains: aesthetic concern (AC; three items), psychological impact (PI; six items), social impact (SI; eight items), and dental self‐confidence (DSC; six items). The PIDAQ is used globally to evaluate the effect of dental aesthetics on the psychosocial status of young adults and connect the scores to factors of interest [21]. However, to the best of our knowledge, this questionnaire has not been used in any Jordanian population. This study aimed to investigate the association between self‐perceived and self‐reported dental aesthetic evaluation and both academic and social performance among Jordanian dental students.

## 2. Methods

### 2.1. Study Type and Setting

Currently, two schools offer undergraduate dental training in Jordan: The University of Jordan (JU) and the Jordan University of Science and Technology (JUST). Data were collected through an anonymous, self‐administered online questionnaire. The data collection window was opened in May 2022 and closed in July 2022. All responses were stored securely in a password‐protected Google Drive folder accessible only to the research team.

### 2.2. Participants

Ethical approval was obtained from the Ethical Review Board of the Deanship of Scientific Research at the JU (Decision No. (35‐2022)). All registered undergraduate dental students at UJ and JUST were eligible to participate. Inclusion criteria were students enrolled in preclinical (1st to 3rd year) and clinical (4th and 5th year internship) phases. A total of 400 participants were invited. Students who declined participation (*n* = 29) were excluded.

### 2.3. Instrument and Variables

This cross‐sectional study relied on the PIDAQ questionnaire to assess students’ perceived dental aesthetics and their psychosocial impact. No clinical assessments or external benchmarks were included, and data are based on self‐reported perceptions. As such, results reflect participants’ subjective experiences rather than objective measurements of dental appearance. Potential confounding variables such as socioeconomic status, mental health, or personality traits were not measured in this study. The questionnaire was filled out by all participants either in person or online via Google Forms, which was shared through social media platforms such as WhatsApp and Instagram, after obtaining informed consent.

The questionnaire had five segments; the first segment assessed sociodemographic information (age, sex, academic year, and history of repeating a study year. All items were self‐reported). The second segment identified the presence of existing dental defects that jeopardise aesthetic appearance. Participants were asked to answer yes or no questions for any of the following: anterior teeth loss, anterior teeth caries, anterior teeth chipping, cracks or spots, central diastema, maxillary anterior teeth crowding, faulty anterior restorations). Participants were categorised into two groups: (1) no compromised aesthetics (answers were No to all questions in this segment) and (2) compromised aesthetics (answers included a Yes to one or more of the questions). The third segment asked participants to evaluate their satisfaction with some dental aesthetic factors including teeth size, colour, shape, and the overall alignment of the teeth, on a Likert scale. The fourth segment assessed the impact of self‐evaluated teeth aesthetics on social performance and self‐confidence using PIDAQ. PIDAQ scores were treated as continuous variables, while subscale scores (Dental Self‐Confidence, Social Impact, Psychological Impact, Aesthetic Concern) were analysed according to standard scoring guidelines. PIDAQ scores calculated for each subdomain (AC, PI, SI, DSC) on a scale of 1–5, with higher scores indicating greater psychosocial impact. The fifth segment recorded the academic performance scores using GPA and self‐satisfaction with that score.

### 2.4. Academic Outcomes

GPA (categorised), academic satisfaction, and history of repeating a year. Categories for GPA were defined as follows: Pass (2.0–2.49), Good (2.5–2.99), Very Good (3.0–3.59), Excellent (3.6–3.79), and Distinction (3.8–4.0). All variables were collected using the same questionnaire for both groups, ensuring full comparability of measurement across the sample. The PIDAQ scores and academic scores were compared between the two groups. Potential biases include self‐reporting bias, social desirability bias, and selection bias due to voluntary participation. Continuous variables (e.g., PIDAQ total score) were analysed as means, while ordinal variables (GPA category, satisfaction) were analysed as frequencies.

### 2.5. Statistical Analysis

Sample size calculation was done after running a pilot study and calculating power analysis using SPSS with 95% confidence intervals and 5% as a margin of error. The minimum sample size was 349 students.

Data were analysed using IBM SPSS Statistics (Version 26) and GraphPad Prism (Version 8.1.1(300)). Descriptive statistics (frequencies, percentages, means, and standard deviations) were used to summarise the data, and inferential statistics, including chi‐square tests, were used to compare categorical variables, such as GPA categories and aesthetic status, while independent *t*‐tests were employed for continuous outcomes (PIDAQ scores). The questionnaire was completed by all participants who consented to participate.

## 3. Results

### 3.1. Demographics

A total of 400 students were invited to participate, of whom 370 completed the survey, yielding a response rate of 92.5%; all forms were completed, full data set can be found in Supporting Information Table S1. Among participants, 228 were females (77.8%) and 82 males (22%). Jordanian students’ percentage was 87.3% (323 students), while 12.7% of participants were non‐Jordanian (47 students). The distribution of students across the study years is shown in Table 1.

**Table 1:** Demographics of students participating in the study.

Parameter studied	*N* (370)	*N* (%)
University		
JU	284	76.8
JUST	86	23.2
Gender		
Female	228	77.8
Male	82	22
Nationality		
Jordanian	323	87.3
Non‐Jordanian	47	12.7
Study level		
1^st^ year	88	23.8
2^nd^ year	33	8.9
3^rd^ year	160	43.2
4^th^ year	44	11.9
5^th^ year	30	8.1
Intern	15	4.1%

Among the parameters studied as contributors to poor facial and dental aesthetics included: anterior teeth loss visible to others while speaking, smiling, or laughing, chipping, caries, faulty restorations, crowding, and anterior diastema. The data showed that more than half of the students (52.7%) have previously had treatments to enhance the aesthetic appearance of their teeth and smiles. Moreover, around 65.4% of students reported they will enhance their dental aesthetics through dental treatments in the future. Other contributors to undermined dental aesthetics are shown in Table 2.

**Table 2:** Prevalence of anterior tooth problems and dental treatment intentions among participants.

Number of students with	*N* (370)	(%)
Anterior tooth loss	19	5.1
Chipping of anterior teeth	49	13.2
Crowding of anterior teeth	37	10
Apparent caries on anterior teeth	22	5.9
Faulty restoration on anterior teeth	24	6.5
Existence of anterior teeth cracks	45	12.2
Presence of Diastema	44	11.9
Presence of spots on the anterior teeth	69	18.6
Gummy smile	54	14.6
Number of students who sought dental treatment	195	52.7
Students who are going to seek dental treatment in the near future	242	65.4

Student’s satisfaction with the aesthetic appearance of eight parameters was measured using a 1 – 10 scale, where “1” is for the least satisfied and “10” is the most satisfied. The parameters used were the teeth size, teeth colour, gum shape, lip shape, teeth alignment, overall dental aesthetics, the appearance of the smile, and overall character of the face.

Most students who participated in this study reported being satisfied and extremely satisfied with their dental aesthetics. As shown in Table 3, the majority of students were satisfied with their teeth size (85.2%), lip shape (83.9), gum Shape (83%), and overall facial character (80.2). A lower percentage of students reported being satisfied with their teeth alignment (77.7%), smile appearance (75.7%), and overall aesthetic satisfaction (74%). On the other hand, 60.2% of dental students were satisfied with their teeth colour.

**Table 3:** Distribution of students’ satisfaction levels with facial and dental aesthetic features.

Aesthetic feature	Extremely unsatisfied (%)	Unsatisfied (%)	Satisfied (%)	Extremely satisfied (%)
Teeth size	1.9	7.9	14.3	76
Teeth colour	6.8	17.2	40.6	35.3
Gum shape	4.3	8.7	13.3	73.8
Lip shape	1.9	7.5	19.8	70.9
Teeth alignment	5.4	9.5	23.3	62
Overall aesthetic satisfaction	4.7	12.2	25.9	57.1
Smile appearance	5.5	9.2	27	58.4
Facial characters	1.1	8.7	32.4	57.7

### 3.2. Social Performance

To evaluate the impact of teeth aesthetics on self‐confidence, the study integrated the Psychosocial Impact of Dental Aesthetic Questionnaire (PIDAQ) form, using its four subscales. The subscales represent four domains: aesthetic concern (AC; five items), psychological impact (PI; 4 items), social impact (SI; six items), and dental self‐confidence (DSC; three items). Before calculating scores, collected data were separated into two groups: students who have their aesthetic character not compromised by any of the aforementioned factors in Table 2 and students who have one or more factors that affect their aesthetic appearance of anterior teeth. PIDAQ scores (out of five) were calculated as means using GraphPad Prism. Pair‐wise comparisons using an unpaired *t*‐test were used to compare the difference in PIDAQ scores of similar items between groups. As shown in Figure 1, there was a statistically significant difference between several items of all four subdomains. Regarding the social impact scale, students who have one or more factors that compromise their dental aesthetics were holding back when smiling and holding hands to hide their smiles during social interaction (*p*  < 0.03) (Figure 1A). Students who have compromised aesthetics were also more dentally self‐conscious; these students were less proud to show their teeth and were less pleased to see their teeth in the mirror, videos, and pictures (*p*  < 0.03) (Figure 1B). Compromised dental aesthetics also had an impact on the psychology of the students affected. Students with compromised aesthetics were significantly resentful of smiles that are more attractive and were more distressed when they saw other people’s teeth (Figure 1C). Students with compromised aesthetics were also more likely to have aesthetic concerns; they were somewhat unhappy about the appearance of their teeth, more concerned about the appearance of their teeth when meeting new people, and they thought most people have nicer teeth than theirs (*p*  < 0.03) (Figure 1D).

Figure 1Results of the PIDAQ score four subdomains showing pair wise comparisons of score means (out of 5) between compromised aesthetics (CA) participants and participants who did not report any factor that affect anterior teeth aesthetics. Asterisks indicate statistical significance based on the Chi‐square test:  ^∗^ *p* < 0.03;  ^∗∗^ *p* < 0.001. (A) Social impact, (B) dental self‐confidence, (C) psycological impact, and (D) aesthetic concern.

**Figure:**
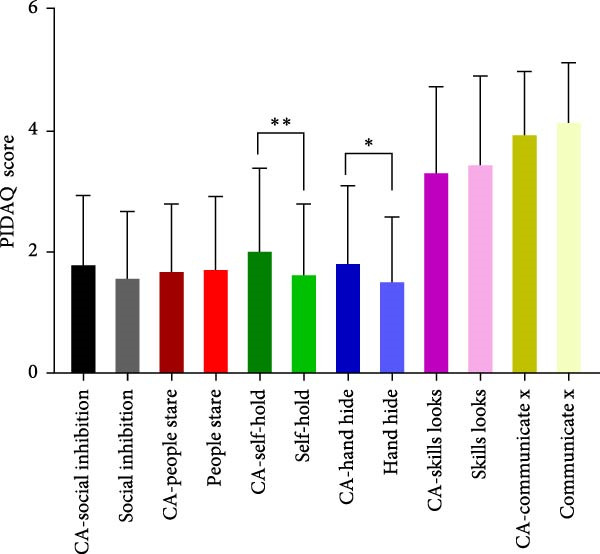
(A)

**Figure:**
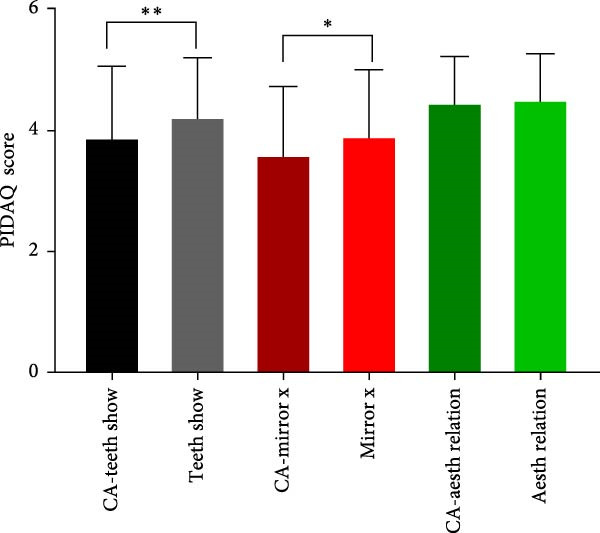
(B)

**Figure:**
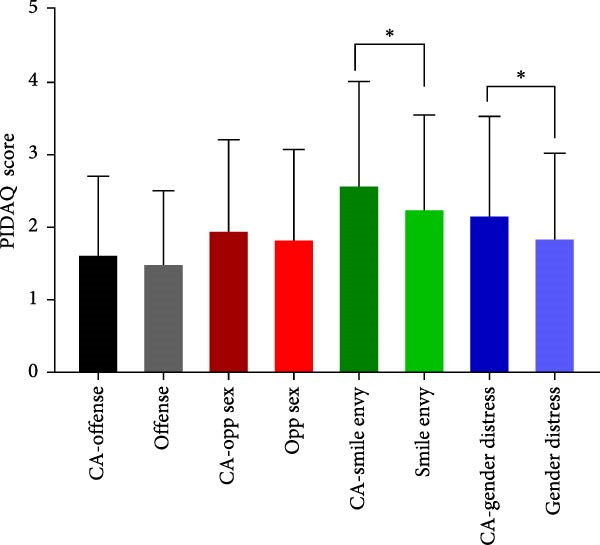
(C)

**Figure:**
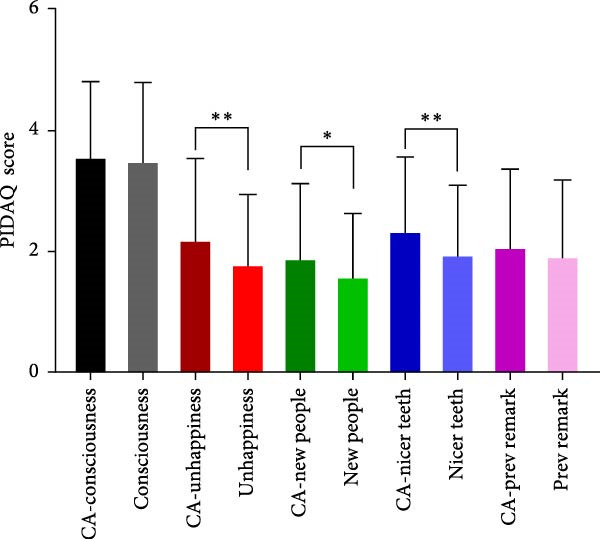
(D)

### 3.3. Academic Performance

To assess academic performance, academic variables including students’ GPA, year repetition, and self‐reported academic satisfaction were collected and analysed. Frequencies for each variable were calculated for students with noncompromised versus compromised dental aesthetics, and differences between groups were evaluated using a chi‐square/asymptotic significance test through SPSS.

Table 4 shows the frequency distribution of both groups regarding GPA, repeating a year, and academic satisfaction. Students with no compromised aesthetic appearance generally had higher GPAs and reported greater satisfaction with their academic achievement, while higher frequencies of lower GPAs and dissatisfaction with academic performance were seen in the group with compromised aesthetics. Significant differences (*p*  < 0.05) were observed between the two groups for GPA and academic satisfaction.

**Table 4:** Academic performance variables distribution between students (no compromise to aesthetics and compromised aesthetics).

Compared groups							Chi‐square	df	Asymp. sig.
Nocompromised aesthetics	University grade ^∗^	Pass	Good	V. good	Excellent	Distinction	95.069^a^	4	0.000
		1.5%	9.9%	43.5%	37.4%	7.6%			
	Year repeat	Yes			No		9.420^a^	4	0.051
		11.5%			88.5%				
	Academic satisfaction	Extremely unsatisfied	Unsatisfied	Neutral	Satisfied	Extremely satisfied	85.450^a^	4	0.000
		0.8%	9.9 %	39.7%	38.9%	10.7%			

Compromised aesthetics (CA)	University grade	Pass	Good	Very good	Excellent	Distinction	83.466^a^	4	0.000
		2.9%	13.8%	49.0%	27.2%	7.1%			
	Year repeat	Yes			No		9.496^a^	4	0.050
		12.6%			87.4%				
	Academic satisfaction	Extremely unsatisfied	Unsatisfied	Neutral	Satisfied	Extremely satisfied	33.084^a^	4	0.000
		5.4%	13.4%	33.5%	33.9%	13.8%			

^a^Pearson Chi‐square test. Used to assess associations between variables Significance is shown in the Asymp. sig. column (*p* < 0.05).

^∗^GPA categories: Pass (2–2.49/4), Good (2.5–2.99/4), Very good (V. good) (3–3.59/4), Excellent (3.6–3.79/4), and Distinction (3.8–4/4).

## 4. Discussion

It is well documented that dental and facial aesthetics play a great role in the career development and psycho‐social well‐being of young individuals [22, 23]. This is due to many factors, but mainly dental aesthetics, how individuals perceive themselves, and how others perceive their smiles within sociocultural and professional contexts [24, 25]. In Jordan, the study of aesthetics and smile/teeth proportions is taught in the first year of dentistry courses onwards, which increases the standards and self‐awareness of aesthetics appearance for students throughout their study years. Few studies have addressed how aesthetics concerns can affect the psychological well‐being of dental students. In this study, we aimed to evaluate the differences in PIDAQ scores between students who identified their smiles as flawless and students who had minor‐to‐major aesthetic problems.

Two factors had the lowest levels of self‐perceived dental aesthetics satisfaction; teeth colour and overall malalignment, which is consistent with a previous study [26]. As in Ellakany’s study, teeth alignment and tooth colour were the two highest contributors to dissatisfaction with one’s smile appearance. Thus, the high percentage of students reporting that they will seek dental treatment to improve their aesthetics may be attributed to the low satisfaction with teeth colour or alignment. This is not a new phenomenon, as it was observed before in a study in 2011, which concluded that patients are willing to undergo dental treatment to improve tooth colour, which would consequently lead to higher levels of aesthetic satisfaction [27].

PIDAQ scores results revealed a significant difference in all four subdomains of the questionnaire between students with compromised aesthetics and students with noncompromised aesthetics. This finding aligns with existing literature, where several studies have demonstrated that dissatisfaction with dental appearance is strongly associated with negative psychosocial outcomes, such as lowered self‐esteem, social avoidance, and increased self‐consciousness [21, 28]. For instance, Klages et al. [20] found that individuals with malocclusion reported higher psychosocial impact scores, indicating that even minor dental aesthetic issues can influence social interactions and emotional well‐being. Similarly, studies on young adults in health sciences have shown that dental aesthetics significantly affect social confidence and interpersonal communication [29].

Our results indicate that perceived dental aesthetic characteristics are associated with differences in self‐reported social interaction and social confidence among dental students, skills that are essential for academic and clinical settings. The fact that even slight deterioration in teeth appearance can affect many aspects of confidence and communication may be due to dental students’ heightened awareness of oral health and aesthetics, given their education and clinical exposure. This heightened sensitivity may be associated with a stronger awareness of oral health and aesthetics in dental students compared to the general population [30]. Furthermore, dental students’ regular involvement in examining, diagnosing, and managing dental and maxillofacial conditions may create a deeper personal and professional association with oral health standards, leading to internalised expectations of perfection.

This internal pressure may help explain the observed differences in academic performance indicators. A significant difference was noted between the GPA scores and academic satisfaction levels of students with and without aesthetic concerns. This could be attributed to the fact that success in dental school relies not only on academic ability but also on effective social communication, self‐confidence, and professional self‐awareness. Previous studies have shown that students who perceive their dental aesthetics positively tend to report higher levels of self‐confidence [18], while poor dental alignment has been associated with reduced academic performance in younger populations [31]. While these findings show an association, the cross‐sectional design of this study does not allow causal inferences, and multiple factors (e.g., prior academic achievement, socioeconomic background, personality traits) may contribute to these differences.

Together, these factors illustrate why dental students may experience disproportionately greater psychological and social impact from even subtle aesthetic deviations. This highlights the need for supportive measures in dental education, such as psychological counselling and open discussions about aesthetic self‐perception, to mitigate the emotional toll and promote healthier self‐image among future dental professionals.

While this study provides several outcomes regarding linking dental aesthetics to academic and social performance of dental students it has its limitations. First, its cross‐sectional design limits the ability to establish causal relationships between dental aesthetics and academic or social performance. Second, the use of self‐reported data may introduce response and social desirability biases. Third, although the sample included students from both dental schools in Jordan, the results may not be generalisable to dental students in other countries or academic environments. Lastly, the survey did not include clinical assessment of dental aesthetics, which might have provided an objective comparison to self‐perceived dental concerns.

Additionally, unmeasured confounding variables such as socioeconomic status, prior dental treatment, mental health, and personality traits could have influenced students’ self‐perceptions and academic or social outcomes. These limitations mean that while we report associations, we cannot conclude that dental aesthetics determine social or academic performance.

## 5. Conclusion

The social and academic performance of dental students in Jordan, was associated with the self‐reported aesthetic quality of their dental features. Teeth size, colour and alignment were the main factors linked to students’ perception of their success as dental students or future dentists, and their overall satisfaction with their academic journey. Based on these findings, it is recommended that dental schools in Jordan consider implementing psychological and peer support systems for students who experience dental aesthetic concerns. Incorporating discussions on self‐image, communication skills, and emotional resilience into dental education may also help mitigate potential psychosocial impacts of perceived aesthetic deficiencies.

Future studies are warranted to explore whether enhancing dental aesthetics can improve academic outcomes and social confidence among students with aesthetic concerns, while considering other influencing factors such as socioeconomic background and personality traits.

## Ethics Statement

The study was conducted in accordance with the Declaration of Helsinki and approved by the Institutional Review Board (IRB) of the University of Jordan (decision No. 35‐2022). Informed consent was obtained from all subjects involved in the study.

## Disclosure

This research was partly presented at the IADR Meeting ‐ 2023 Jordanian Section as an oral presentation in the oral session held on June 23, 2023.

## Conflicts of Interest

The authors declare no conflicts of interest.

## Author Contributions

**Aseel Sharaireh:** conceptualisation, methodology, formal analysis, investigation, writing – original draft, writing – review & editing. **Eman Hassuneh:** conceptualisation, methodology, investigation, writing – review & editing. **Hiba Nasser:** conceptualisation, methodology, investigation, writing – review & editing. **Nesreen Salem:** methodology, investigation. **Sanaa Aljamani:** methodology, investigation. **Hassan Kaabi:** formal analysis, writing – review & editing. **Faleh Sawair:** formal analysis, writing – review & editing. **Mohammad AL-Rabab’ah:** supervision, project management, writing – review & editing.

## Funding

No external funding, apart from the support of the authors’ institution, was available for this study.

## Supporting Information

Additional supporting information can be found online in the Supporting Information section.

## Supporting information


**Supporting Information** Full raw data where all participants’ answers are recorded and used in the analysis that followed are available in “Table S1”. The first row contains the question stem used in the survey and the following rows are the answers collected form each participant, names are anonymous for ethical considerations.

## Data Availability

All data generated and analysed during this study are included in the Supporting Information of this article.
